# Harnessing Tumor Cell‐Derived Exosomes for Immune Rejection Management in Corneal Transplantation

**DOI:** 10.1002/advs.202409207

**Published:** 2024-11-14

**Authors:** Jieru Yang, Huanmin Kang, Yingyi Liu, Shan Lu, Huihui Wu, Bikui Zhang, Yan He, Wenhu Zhou

**Affiliations:** ^1^ Xiangya School of Pharmaceutical Sciences Central South University Changsha Hunan 410013 China; ^2^ Department of Ophthalmology West China Hospital Sichuan University Chengdu Sichuan 610041 China; ^3^ Beijing Tongren Eye Center Beijing Tongren Hospital Capital Medical University Beijing Key Laboratory of Ophthalmology & Visual Sciences Beijing 100730 China; ^4^ Department of Pharmacy The Second Xiangya Hospital Central South University Changsha 410011 China; ^5^ Key Laboratory of Biological Nanotechnology of National Health Commission Changsha Hunan 410008 China

**Keywords:** angiogenesis, B16‐F10 melanoma, corneal transplantation, exosomes, JAK2, myeloid‐derived suppressor cells

## Abstract

Transplantation remains the definitive treatment for end‐stage organ failures, but its efficacy is frequently compromised by immune rejection. This study introduces a novel strategy by utilizing tumor‐derived exosomes from B16‐F10 melanoma cells (B16‐Exo), diverging from the conventional use of immune cell‐derived exosomes, to alleviate post‐transplantation immune rejection. Utilizing murine corneal transplantation as a model, it is demonstrated that B16‐Exo significantly reduces immune rejection, evidenced by decreased corneal opacity, neovascularization, and immune dysregulation, while enhancing postoperative survival. Proteomic analyses reveal differential expression of pivotal proteins in B16‐Exo, notably the JAK2 protein within the JAK‐STAT signaling pathway, which has been mechanistically demonstrated to amplify the activity of myeloid‐derived suppressor cells (MDSCs) and inhibit T cell proliferation. These findings demonstrate the significant immunomodulatory effect of B16‐Exo in transplant immunology, supporting the continued exploration of tumor‐derived exosomes as a platform to uncover novel immunosuppressive mechanisms in transplantation.

## Graphical abstract

This study introduces a novel method to reduce post‐transplant immune rejection using tumor‐derived B16‐F10 melanoma cell exosomes. Employing murine corneal transplantation as a model, B16‐Exo is shown to significantly decrease immune rejection and enhance survival, with proteomic analysis uncovering the critical role of JAK2 in modulating immune cell activity.

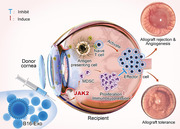

## 1 Introduction

In organ and tissue transplantation, effective management of immune rejection stands as a formidable challenge, centered on the recipient's response to allogeneic grafts.^[^ ^1^ ^]^ Immune rejection following transplantation leads to a multifaceted immune cascade where in host immune cells recognize and initiate responses against alloantigens present in the transplanted tissue. T cells play a central role by attacking transplanted tissues and coordinating other immune responses, exacerbating tissue injury.^[^ ^2^, ^3^ ^]^ Thus, effectively managing immune rejection is crucial for better transplantation outcomes. Presently, immune rejection management heavily relies on prolonged administration of immunosuppressive agents. While these pharmacotherapies significantly limit immune rejection, they also present a series of adverse effects, including heightened susceptibility to infections, metabolic derangements, and neurologic complications.^[^ ^4^, ^5^, ^6^ ^]^ Hence, the development of novel immune regulatory strategies holds immense promise in enhancing the clinical efficacy of organ or tissue transplantation.^[^ ^7^ ^]^ Such strategies not only necessitate effective attenuation of immune rejection^[^ ^8^, ^9^, ^10^ ^]^ but also provide enhancements in long‐term graft survival rates, thereby affording patients safer therapeutic outcomes and broadening the therapeutic vistas of transplantation.

In recent years, exosomes have emerged as compelling candidates for immune modulation. Exosomes, encompassing nanoscale vesicles discharged by cells, serve as carriers of bioactive molecules such as proteins, lipids, and nucleic acids, facilitating intercellular signaling within the immune system.^[^ ^11^, ^12^ ^]^ Indeed, specific subsets of exosomes have been implicated in regulating immune cell function, cytokine dynamics, and immune responses, with their therapeutic potential demonstrated across diverse immune‐related pathologies.^[^ ^13^ ^]^ The immunomodulatory properties of exosomes exhibit context‐dependent variability depending on their cellular origins and application circumstances.^[^ ^14^ ^]^ The immunomodulatory activities of exosomes have been explored in the management of corneal transplantation. For example, exosomes derived from human induced pluripotent stem cells,^[^ ^15^ ^]^ bone marrow mesenchymal stem cells,^[^ ^16^ ^]^ human adipose tissue, and umbilical cord stem cells^[^ ^17^ ^]^ have shown potential in combating corneal transplant rejection. However, their high cost limits their widespread application. Exosomes from various immune cells, including myeloid‐derived suppressor cells (MDSCs) and dendritic cells (DCs), exhibit diverse immunomodulatory effects depending on the immune cell's activation state and functional role. While exosomes from some immune cells may promote immune responses or support tissue repair, others, such as MDSC‐derived exosomes, play a more direct role in immune suppression.^[^ ^18^, ^19^, ^20^ ^]^

Significant research has demonstrated the crucial role of tumor‐derived exosomes in fostering an immunosuppressive microenvironment, effectively serving as natural tools for immune evasion by tumor cells.^[^ ^21^, ^22^, ^23^ ^]^ Tumor exosomes exert inhibitory effects on T cell activity, suppress T cell receptor signaling, and thereby attenuate T cell‐mediated immune responses.^[^ ^24^ ^]^ Given the conjecture immunosuppressive attributes of tumor exosomes, conjecture arises regarding their potential utility in transplant immunology, although related investigations remain nascent. Compared to exosomes from other cells, tumor‐derived exosomes deliver immunosuppressive molecules such as PD‐L1 and FasL more effectively, inhibiting the activity of T cells and Natural Killer cells.^[^ ^25^, ^26^, ^27^ ^]^ They construct an inhibitory immune microenvironment by promoting the proliferation and activation of MDSCs and regulatory T cells (Tregs).^[^ ^28^, ^29^ ^]^ Additionally, exosomes from tumor cells are more easily obtainable and produced in higher quantities than those from other cell types,^[^ ^30^ ^]^ which provides convenience for experimental research.

**Scheme 1:**
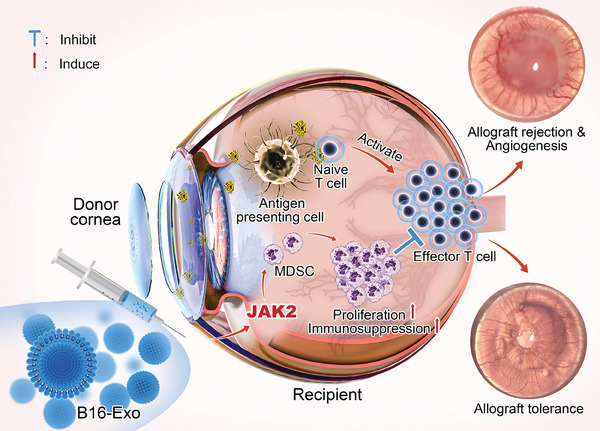
Scheme of B16‐Exo Mediated Immunoregulation in Corneal Transplantation: The illustration represents the subconjunctival injection of B16‐Exo, which modulate immune responses during corneal transplantation. B16‐Exo targets the JAK2 pathway to promote the proliferation and immunosuppressive activity of myeloid‐derived suppressor cells (MDSCs), inhibiting the activation and proliferation of T cells. The presence of antigen‐presenting cells (APCs) and naive T cells are also shown, indicating the potential point of T cell proliferation that B16‐Exo may suppress. This schematic captures the therapeutic strategy of using B16‐Exo to prevent graft rejection and prolong transplant survival by modulating the immune response in the ocular environment. Exo: Exosome, Teff: Effector T cell.

Based on the above‐mentioned facts, we utilized exosomes derived from B16‐F10 melanoma cells (B16‐Exo) to explore their therapeutic potential in regulating immune rejection during transplantation (**Scheme** **1**). Employing murine corneal transplantation as a model system, we substantiate the immune‐repressive effects of B16‐Exo following transplant rejection, accompanied by improvements in postoperative survival. The unique attributes of the cornea, including its transparency, and avascularity render it an ideal experimental platform for investigating immune rejection, process of neovascularization, and its modulation.^[^ ^31^, ^32^ ^]^ Our findings affirm the sustained retention of B16‐Exo in the ocular milieu for over 6 days post‐subconjunctival administration, concomitant with notable reductions in corneal opacity, neovascularization, and immune infiltration. Proteomic analyses and mechanistic elucidations demonstrate the immunomodulatory properties of B16‐Exo, mediated via the JAK2 protein constituent, which exerts regulatory effects on the JAK‐STAT signaling axis, potentiating the activity of myeloid‐derived suppressor MDSCs while diminishing T cell proliferation. This study demonstrates the therapeutic efficacy of B16‐Exo in inhibiting corneal transplant immune rejection, thereby providing a novel strategy for the development of immune‐modulatory agents in organ transplantation management.

## 2 Results and Discussion

### 2.1 Therapeutic Efficacy of B16‐Exo in Reducing Immune Rejection in Allogeneic Corneal Transplantation

**Figure 1:**
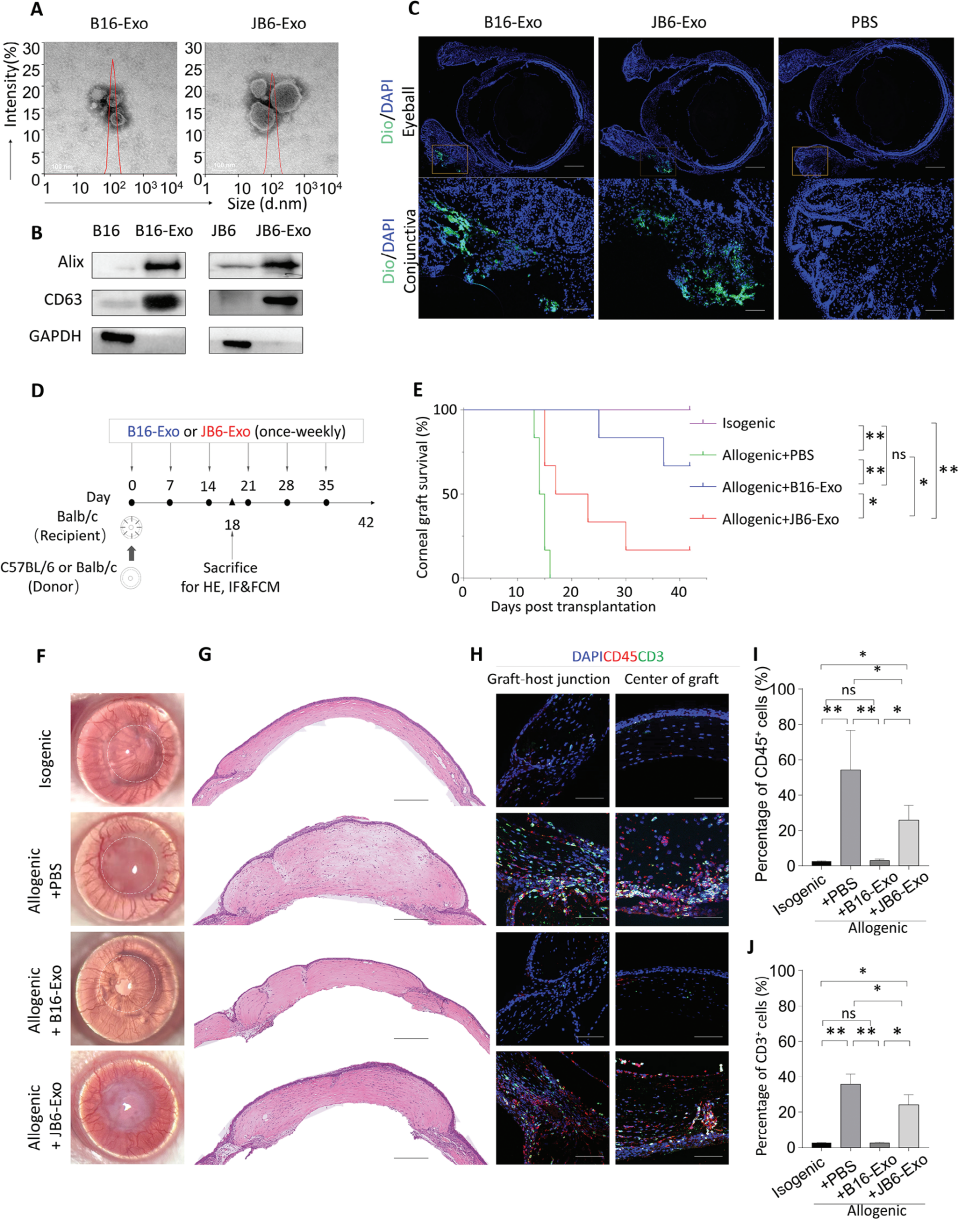
(A) Representative transmission electron microscopy (TEM) images and size distributions of B16‐Exo and JB6‐Exo. Scale bar: 100 nm. (B) Western blotting analysis of exosome markers (Alix, CD63, GAPDH) in different exosomes. (C) Tracking of exosomes labeled with Dio injected into mice receiving corneal transplantation via subconjunctival injection. Distribution on day 6 post‐injection is shown. Scale bar: 500 µm (original), 100 µm (enlarged). (D) Schematic representation of corneal transplantations performed on day 0, with exosomes or PBS administered conjunctively once a week until day 35. Corneas were observed every other day until day 42. Mice were randomly selected on day 18 and sacrificed for hematoxylin‐eosin (HE) staining, immunofluorescence staining (IF), and Flow Cytometry (FCM). (E) Kaplan–Meier survival curves of isografts and allografts with indicated administration (*n* = 6/group). (F) Slim‐lamp images (front view) of graft‐bearing corneas on day 18. Dashed circles outline the grafts. (G) HE staining of corneal grafts on day 18 post‐transplant. Scale bar: 200 µm. (H) Immune cells (CD45^+^) and T cells (CD3^+^) stained in the graft‐bearing corneas. Scale bar: 100 µm. (I) Percentage of CD45^+^ immune cells in the whole corneal graft. (J) Percentage of CD3^+^ T cells in the whole corneal graft. Bars in (I) and (J) represent mean ± SD (*n* = 3).

We utilized B16‐F10 melanoma cell‐derived exosomes (B16‐Exo) as the experimental model, along with JB6 Cl41 normal epidermal cell exosomes (JB6‐Exo) serving as the control. Exosomes were isolated from the supernatants of B16‐F10 and JB6 Cl41 cell cultures employing gradient centrifugation, yielding B16‐Exo and JB6‐Exo, respectively. Transmission electron microscopy (TEM) examination illustrated the typical spherical morphology of these exosomes (**Figure** **1**A), with a predominant size distribution ≈100 nm, consistent with dynamic light scattering (DLS) measurements. Western blot (WB) analysis further corroborated the presence of extracellular vesicle markers CD63 and Alix (Figure 1B), validating the successful isolation of exosomes.

We assessed the biocompatibility of B16‐Exo within the ocular environment by performing cytotoxicity assays on corneal and conjunctival epithelial cells, which showed no adverse effects at any of the concentrations tested. (Figure S1, Supporting Information). To assess the distribution and persistence of exosomes within the ocular area, fluorescein‐labeled (Dio) B16‐Exo and JB6‐Exo were administered via subconjunctival injection in allogeneic mice. Remarkably, fluorescence signals persisted in the cornea and conjunctiva even after 6 days post‐injection (Figure 1C), indicative of the long‐term retention capability of exosomes within ocular tissues. This feature not only suggests the potential for reducing the frequency of in vivo administration but also augments patient compliance. Consequently, a weekly injection regimen was adopted for subsequent studies.

Subsequent investigations aimed to evaluate the therapeutic efficacy of the two types of exosomes in a murine model of allogeneic corneal transplantation, as showed in Figure 1D. Untreated allogeneic mice exhibited transplant rejection and experienced mortality prior to the designated observation endpoint, with an average survival period of 15 ± 1.0 days. Administration of JB6‐Exo exhibited modest efficacy, with mortality occurring subsequent to the second dose (Day 14), and a survival rate of merely 20% over a 42‐day period. In stark contrast, treatment with B16‐Exo significantly prolonged murine graft survival, yielding a survival rate of 70% within 42 days (Figure 1E). Notably, corneal transparency, a pivotal indicator for successful transplantation, exhibited substantial enhancement in the B16‐Exo group compared to controls by day 18 (Figure 1F). H&E staining of corneal stroma further validated the significant reduction in corneal stromal edema in the B16‐Exo group, with restoration to levels akin to isogenic mice (Figure 1G).

Given that allograft corneal transplantation rejection involves an inflammatory imbalance stemming from excessive immune system activation, we assessed the degree of immune cell infiltration (CD45^+^) and T cells (CD3^+^) in corneal tissue via immunohistochemical staining. Results demonstrated that treatment with B16‐Exo markedly diminished the infiltration of these immune cells (Figure 1H), with fluorescence intensity akin to isogenic controls, underscoring the suppressive effect of B16‐Exo on immune cell infiltration. Conversely, mice treated with JB6‐Exo exhibited notable immune cell infiltration, indicative of weak immune regulatory capacity. Statistical analyses further corroborated a significant reduction in the number of CD45^+^ and CD3^+^ cells in the B16‐Exo treatment group (Figure 1I,J), thereby highlighting the potential of B16‐Exo in modulating immune‐mediated transplant rejection. Consequently, B16‐Exo emerges as a promising therapeutic agent capable of preserving corneal transparency, modulating local immunity, and extending the survival period in allogeneic corneal transplantation, thus underlining its potential utility in transplant medicine.

### 2.2 Inhibition of Angiogenesis by B16‐Exo Following Corneal Transplantation

**Figure 2:**
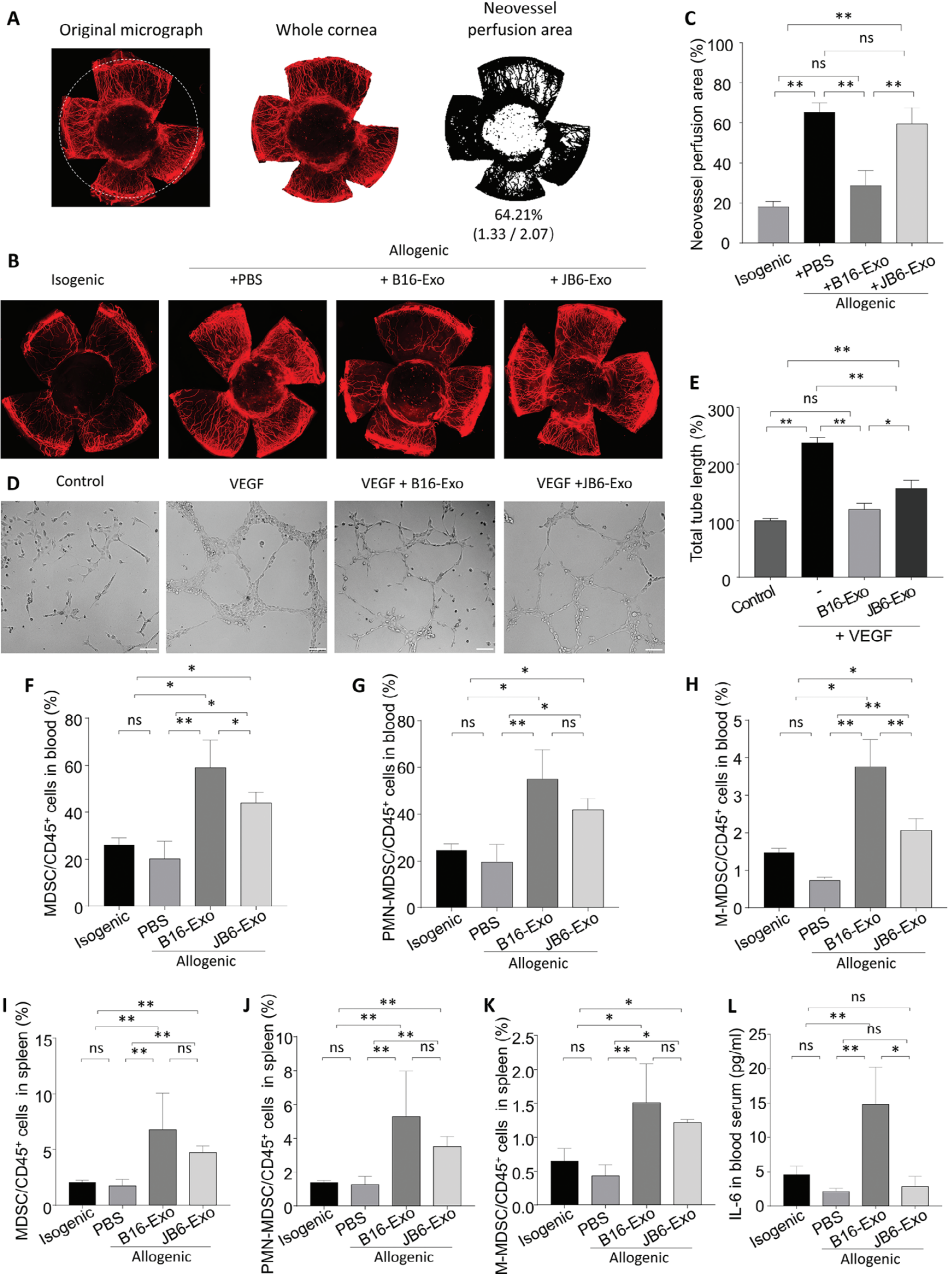
(A) Image processing for neovascularization assessment in graft‐bearing corneas. The original graft‐bearing cornea was delineated by a dashed circle (left), and the entire corneal area was captured (middle). The processed neovascularization area is presented on the right. (B) Representative immunofluorescent micrographs of CD31 staining (for neovessels) in graft‐bearing corneas on day 18 post‐transplant. (C) Quantification of neovascularization area from Figure 2B. Bars represent mean ± SD (*n* = 3/group). (D) Representative images of tube formation in HUVECs treated with VEGF combined with JB6‐Exo or B16‐Exo, bar = 100 µm (E) Quantitative analysis of tube formation. (F–H) Normalized percentages of MDSCs, granulocytic MDSCs (PMN‐MDSCs), and monocytic MDSCs (M‐MDSCs) accounting for CD45^+^ immune cells in the blood. (I–L) Normalized percentages of MDSCs, PMN‐MDSCs, and M‐MDSCs accounting for CD45^+^ immune cells in the spleen. (H) Quantification of IL‐6 level in blood serum on day 18 post‐transplant. Bars in (B–H) represent mean ± SD (*n* = 3/group).

After our evaluation of the therapeutic impact of B16‐Exo on immune rejection in corneal transplantation, we examined its effect on neovascularization post‐transplantation. The cornea, being inherently avascular, often exhibits heightened neovascularization during immune rejection episodes.^[^ ^33^ ^]^ This angiogenic response is attributed to the host immune system's assault on allogeneic corneal tissue, triggering inflammation and the subsequent release of angiogenic factors, thereby facilitating endothelial cell migration and proliferation.^[^ ^34^, ^35^ ^]^ Consequently, neovascularization serves as a crucial indicator for gauging the extent of allograft rejection. Visualization of the neovascular network was accomplished through immunofluorescence staining targeting CD31, an endothelial cell marker.^[^ ^36^ ^]^ Subsequent to image processing of the acquired immunofluorescence micrographs, quantitative assessment of the neovascular perfusion area was conducted (**Figure** **2**A). Comparative analysis revealed that following B16‐Exo treatment, neovascularization in allogeneic mice reverted to baseline levels (Figure 2B), thus affirming its inhibitory effect on angiogenesis. Conversely, mice treated with JB6‐Exo displayed a notably elevated corneal neovascularization perfusion area compared to isogenic controls (Figure 2C), indicative of the limited efficacy of JB6‐Exo in inhibiting neovascularization. This observation demonstrates the potential of B16‐Exo as an effective modulator of angiogenesis post‐corneal transplantation, suggesting its utility in mitigating vascular complications associated with allograft rejection. To further investigate the effect of B16‐Exo on angiogenesis, we performed in vitro tube formation assays using HUVECs. The results showed that B16‐Exo significantly inhibited tubular structure formation compared to JB6‐Exo (Figure 2D,E), supporting our in vivo findings that B16‐Exo has a more distinct anti‐angiogenic effect.

### 2.3 Regulation of Bone Marrow‐Derived Suppressor Cells Proliferation and Function by B16‐Exo

Acknowledging the pivotal role of MDSCs in orchestrating corneal allograft tolerance,^[^ ^37^ ^]^ we sought to clarify the regulatory impact of exosomes on MDSC proliferation and function, thereby delving deeper into their mechanistic underpinnings. We first explored the biocompatibility of B16‐Exo with MDSCs using the CCK‐8 assay. After co‐culturing with B16‐Exo for 24 h, the survival rate of MDSCs significantly increased (Figure S2, Supporting Information), indicating that B16‐Exo may promote the survival or proliferation of MDSCs, demonstrating its good biocompatibility. On the 18th day post‐transplantation, blood and spleen specimens were collected from experimental mice for MDSC isolation and subtype characterization (Figure S3, Supporting Information). Analysis revealed that the B16‐Exo treatment group exhibited the highest MDSC content in peripheral blood (Figure 2F), significantly surpassing levels observed in the JB6‐Exo‐treated cohort. This distinction was evident not only in the overall MDSC population but also in the PMN‐MDSC and M‐MDSC subsets (Figure 2G,H). Furthermore, a corresponding augmentation in MDSC levels was noted in the spleen of B16‐Exo‐treated mice, surpassing the efficacy of JB6‐Exo treatment (Figure 2I–K). Hence, compared to JB6‐Exo, B16‐Exo exerted a more pronounced stimulatory effect on MDSC proliferation. As a control, we also tested Tregs, while no significant change in the number of Tregs post‐treatment (Figure S4, Supporting Information), confirming that MDSCs is the primary target of the exosomes in inducing immune tolerance. Concurrently, the level of inflammatory mediator in mouse serum was assessed to gauge the impact of exosomes on MDSC function. Notably, IL‐6, a potent MDSC stimulator, exhibited significant elevation in the B16‐Exo treatment group (Figure 2L). Collectively, these findings reveal the dual capacity of B16‐Exo in promoting MDSC expansion and augmenting their functional repertoire, thereby engendering potent immunosuppressive effects.

### 2.4 Proteomic Profiling Reveals Differential Expression and Potential Functional Significance of JAK2 in B16‐Exo

**Figure 3:**
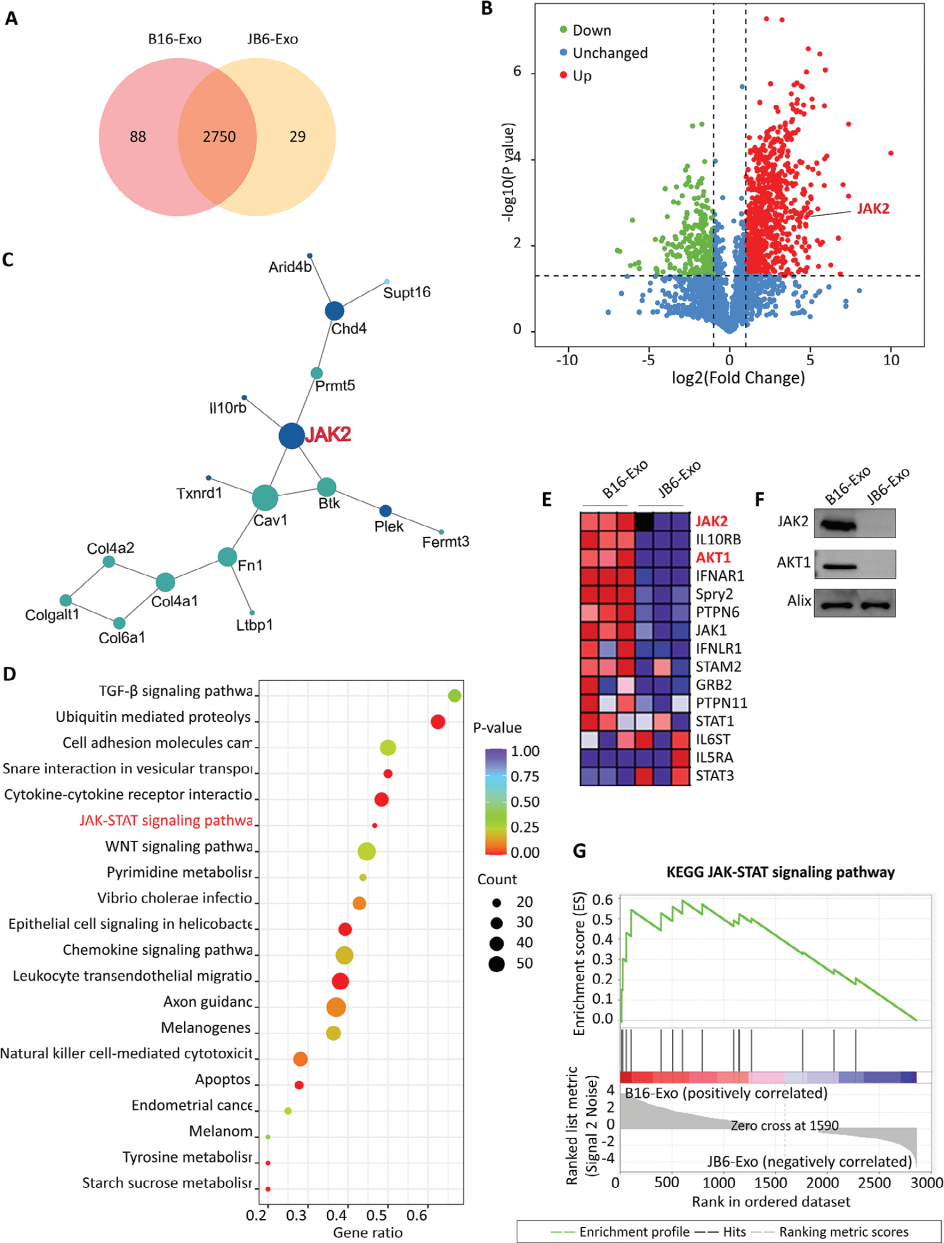
(A) Venn diagram showing the number of proteins shared in B16‐Exo and JB6‐Exo. (B) Volcano map of the differentially expressed protein distribution. JAK2 (−log10 *p* value = 2.73, log2 Fold Change = 4.86) is indicated by black lines. (C) Protein–protein interaction (PPI) network showing JAK2 as the most frequently interacted protein (degree = 4). Intense blue circles and cyan circles represent upregulated and downregulated proteins in the B16‐Exo group, respectively (*p* < 0.05, Fold Change ≥ 2, Top 50 proteins were selected). Other proteins without interaction are not presented. The size of the circles is proportional to the number of notes. (D) KEGG function enrichment analysis of B16‐Exo using the gene set enrichment analysis (GSEA) (top 20 terms). (E) Heatmap showing differentially expressed proteins in the JAK‐STAT signaling pathway based on GSEA. The range of colors (red, pink, light blue, dark blue) indicates the range of expression values (high, moderate, low, lowest). (F) Western blot validation of the JAK2 and AKT1 protein expression levels. (G) Enrichment Score (ES) of the JAK‐STAT Signaling Pathway.

In pursuit of clarifying the underlying mechanisms contributing to the disparate efficacy observed between B16‐Exo and JB6‐Exo in corneal transplantation treatment, we employed proteomic techniques to conduct a comparative analysis of the two exosome types, with a particular emphasis on discerning protein expression disparities and their putative biological implications. Proteomic sequencing unveiled a total of 2750 proteins expressed in both B16‐Exo and JB6‐Exo. Notably, B16‐Exo exhibited exclusive expression of 88 proteins, whereas JB6‐Exo harbored 29 unique proteins (**Figure** **3**A). Differential expression analysis revealed 790 upregulated proteins and 241 downregulated proteins in B16‐Exo (fold change ≥ 2, P < 0.05, Figure 3B). Subsequent protein‐protein interaction (PPI) network analysis pinpointed JAK2 as a central upregulated node (degree = 4), emphasizing its pivotal role within the B16‐Exo proteome (Figure 3C).

KEGG pathway analysis predominantly implicated the gene expression profile of B16‐Exo with the JAK‐STAT pathway, thus reinforcing the notion that JAK2 assumes a critical role in the immune regulatory functions of B16‐Exo (Figure 3D). We conducted a gene set enrichment analysis (GSEA) to identify signaling pathways significantly associated with the proteomic profiles of B16 melanoma‐derived exosomes (B16‐Exo). Visualization of differentially expressed proteins within the JAK‐STAT signaling pathway was facilitated through heatmap analysis (Figure 3E). To validate the proteomic results, we analyzed the expression levels of JAK2 and AKT1 proteins, which are closely related to the JAK‐STAT signaling pathway. These two proteins were significantly upregulated in B16‐Exo but not in JB6‐Exo, consistent with our protein heatmap data (Figure 3F). This further supports the importance of the JAK‐STAT pathway in the immunomodulatory effects of B16‐Exo. The GSEA revealed a notable enrichment of the KEGG JAK‐STAT signaling pathway. As demonstrated in the enrichment plot (Figure 3G), there was a positive correlation with a peak enrichment score observed at the top of the ranked list metric scores for B16‐Exo, indicating a significant over‐representation of pathway‐associated proteins in B16‐Exo compared to JB6‐Exo, which is shown as negatively correlated. Given the established correlation between JAK2 and the augmentation and regulation of MDSC activity,^[^ ^38^ ^]^ it is plausible that B16‐Exo significantly modulates MDSC functionality via the JAK‐STAT signaling pathway. Although KEGG pathway analysis identified TGF‐β signaling pathway as the most enriched pathway, neither B16‐Exo nor JB6‐Exo expressed TGF‐β. Nevertheless, proteins such as SMAD6, ACVR1, ACVRL1, and TGFR1—key components associated with TGF‐β signaling—were enriched within this pathway. Notably, SMAD6 exerts inhibitory control over TGF‐β signaling via a feedback mechanism,^[^ ^39^ ^]^ while ACVR1 and TGFR1 play pivotal roles in mediating TGF‐β‐induced SMAD1/5 phosphorylation.^[^ ^40^ ^]^ Additionally, ACVRL1 assumes significance in endothelial cell function during adult skin vascular remodeling.^[^ ^41^ ^]^ Considering the pivotal role of MDSCs in immune regulation and anti‐angiogenic processes in corneal transplantation, it is conceivable that B16‐Exo exerts a profound influence on MDSC function via the JAK‐STAT signaling pathway, thereby manifesting its therapeutic efficacy in controlling immune rejection in allogeneic corneal transplantation.

### 2.5 B16‐Exo Mediates Expansion and Functions of MDSCs via the JAK2 Pathway

**Figure 4:**
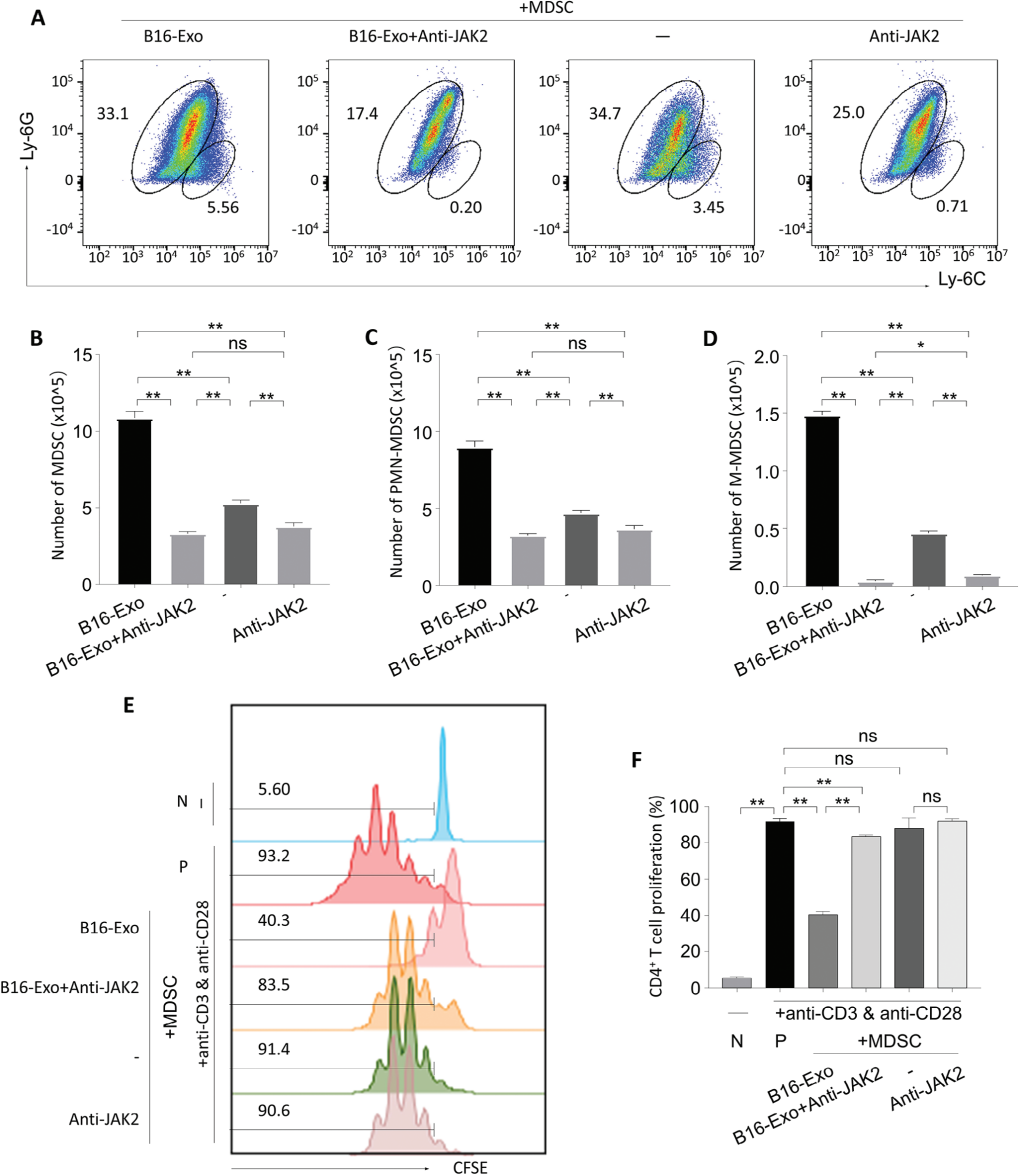
(A) Representative flow cytometry cytograms of MDSC. (B) Number of MDSCs, (C) PMN‐MDSCs, and (D) M‐MDSCs in the presence of MDSC polarization medium (IL‐6 and GM‐CSF) after various treatments. (E) MDSC treated with indicated exosomes in the absence or presence of anti‐JAK2 (AG490, 50 um) for 72 h were collected for coculture with CD4^+^T cells for 5 days. (F) The proliferation of CFSE‐labeled CD4^+^T cells evaluated in Figure 4D is presented.

Subsequent investigations aimed to validate the regulatory effect of B16‐Exo on MDSCs through the JAK‐STAT signaling pathway via anti‐JAK2 inhibitors. MDSCs isolated from murine tibiae were co‐cultured with exosomes under the stimulation of IL‐6 and GM‐CSF for subsequent flow cytometry detection and data analysis (**Figure** **4**A). Comparative analysis revealed a significant increase in the number of MDSCs in the presence of B16‐Exo, consistent with findings from animal experiments, thus further substantiating the proliferative effect of B16‐Exo on MDSCs. Notably, the addition of anti‐JAK2 inhibitors markedly attenuated the activity of B16‐Exo (Figure 4B), resulting in a reduction in the number of MDSCs. Similar trends were observed in the flow cytometry‐based quantitative analysis of PMN‐MDSC and M‐MDSC subsets (Figure 4C,D). These findings collectively demonstrate the prominent role of the JAK2 signaling pathway in mediating the proliferative effects of B16‐Exo on MDSCs.

Furthermore, to clarify whether B16‐Exo modulates T cell proliferation via MDSCs, we co‐cultured B16‐Exo with MDSCs for three days followed by T cells for five days. Results demonstrated that MDSCs treated with B16‐Exo significantly suppressed the proliferation of CD4^+^ T cells (Figure 4E,F). However, the addition of anti‐JAK2 inhibitors mitigated the inhibitory effect on CD4^+^ T cells. Consequently, B16‐Exo selectively modulates MDSC function through the JAK2 pathway, thereby exerting inhibitory effects on T cells. In summary, B16‐Exo robustly enhances the proliferation and immunosuppressive capacity of MDSCs via the JAK2 pathway, thereby effectively regulating the dynamics of T cell proliferation. This mechanistic insight aligns closely with observations of MDSC‐mediated T cell dysfunction during tumorigenesis.^[^ ^42^ ^]^

### 2.6 Role of JAK2 in B16‐Exo‐Induced MDSC Expansion and Functionality In Vivo

**Figure 5:**
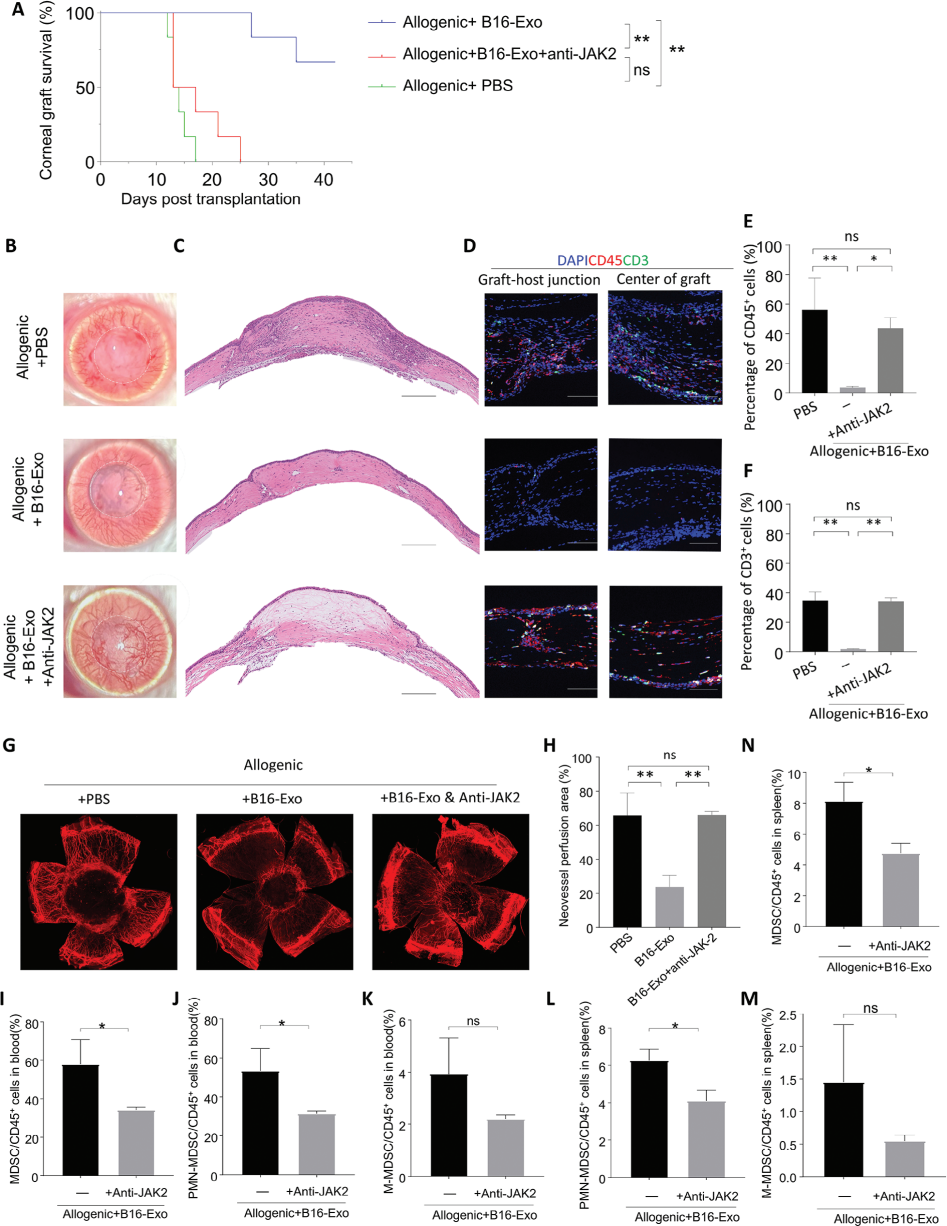
The role of JAK2 in B16 Exo mediated MDSC function and its regulation of corneal transplant rejection. (A) Survival rate curve after transplantation with various treatments. (B) Slim‐lamp images (front view) of graft‐bearing corneas on day 18. Dashed circles outline the grafts. (C) HE staining of corneal grafts on day 18 post‐transplant. Scale bar: 200 µm. (D) Immune cells (CD45^+^) and T cells (CD3^+^) stained in the graft‐bearing corneas. Scale bar: 100 µm. (E,F) Quantitative analysis of CD45 and CD3 immune cells. (G) Corneal images of fluorescently labeled blood vessels. (H) Quantitative analysis results of the neovascularization area. (I–N) The proportion and distribution of MDSCs and their subtypes in the spleen and blood. Bars in (E) and (F) represent mean ± SD (*n* = 3).

To validate the regulation of corneal transplant rejection by B16‐Exo through the JAK2 pathway in vivo, we performed subconjunctival injections of exosomes, followed by continuous injections of JAK2 inhibitor (anti‐JAK2). Compared to the PBS group, B16‐Exo significantly improved transplant survival rates (**Figure** **5**A). However, the addition of anti‐JAK2 significantly reduced the efficacy of B16‐Exo, confirming the crucial role of JAK2 in prolonging survival with B16‐Exo treatment. Observations of corneal transparency and angiogenesis also support these findings. Corneal transparency was higher, and angiogenesis was significantly reduced after treatment with B16‐Exo (Figure 5B). The addition of anti‐JAK2 resulted in partial reversal of these indicators, suggesting that JAK2 inhibitors may counteract the immunosuppressive effect of B16‐Exo.

Additionally, we assessed the degree of corneal interstitial edema. After treatment with B16 Exo, the degree of corneal interstitial edema in mice was significantly reduced (Figure 5C). This therapeutic effect was blocked by anti‐JAK2, highlighting the importance of JAK2 in inhibiting corneal interstitial edema with B16‐Exo. Evaluation of immune cell and T cell infiltration showed that, compared to the PBS group, the infiltration of CD45^+^ immune cells and CD3^+^ T cells in the corneal tissue of mice treated with B16‐Exo was significantly reduced at both the transplant host interface and the transplant center. In contrast, mice in the anti‐JAK2 group showed infiltration levels comparable to the PBS group (Figure 5D–F). In addition to the observed effects on immune cell infiltration, we further explored the impact of B16‐Exo on the local inflammatory response. Focused immunohistochemical studies of the inflammatory cytokine IL‐1β revealed that B16‐Exo treatment significantly diminished IL‐1β expression relative to the PBS control (Figure S5, Supporting Information). Interestingly, the suppression of IL‐1β was notably influenced by the introduction of a JAK2 inhibitor, suggesting that the regulation of IL‐1β by B16‐Exo is predominantly mediated through the JAK‐STAT signaling pathway. Further fluorescence labeling of blood vessels and quantification of vascular area showed that the addition of anti‐JAK2 reversed the reduction in vascular area caused by B16‐Exo (Figure 5G,H). These results confirm that B16‐Exo relies on JAK2 to inhibit corneal transplant rejection in vivo. To explore how B16 Exo affects MDSCs through the JAK‐STAT signaling pathway, we analyzed MDSCs and their subtypes in the spleen and blood of mice. After the addition of anti‐JAK2, the number of MDSCs and PMN‐MDSCs derived from blood and spleen were significantly inhibited (Figure 5I–N), supporting the JAK2‐dependent promotion of MDSC function by B16‐Exo.

## 3 Conclusion

In conclusion, this study demonstrates the therapeutic efficacy of B16‐Exo in ameliorating immune rejection in transplantation. Through murine corneal transplantation models, we clarify the immunomodulatory properties of B16‐Exo, characterized by alleviated corneal opacity, neovascularization, and immune infiltration, alongside improved postoperative survival. Both in vitro and in vivo experiments have revealed the pivotal role of the JAK‐STAT signaling pathway in inhibiting corneal transplant rejection by B16‐Exo. These findings not only enrich our understanding of the immunoregulatory mechanisms of exosomes but also reveal the therapeutic potential of tumor cell exosomes in transplant immunology. Notably, tumor‐derived exosomes mimic and apply tumor immune regulatory mechanisms within the ocular microenvironment, thereby effectively suppressing immune responses. This groundbreaking discovery sets the foundation for utilizing exosomes derived from tumor cells in managing ocular transplant rejection.

## 4 Experimental Section

### Regents

All details regarding the reagents utilized in this research, along with their respective concentrations, are provided in Table S1 (Supporting Information).

### Exosome Isolation and Identification

Exosomes were isolated from the murine melanoma cell line B16‐F10 and murine epidermal JB6 Cl41 cells culture supernatants. The cells were cultured in Dulbecco's Modified Eagle Medium (DMEM; Thermo Fisher Scientific, Massachusetts, USA), supplemented with 10% exosome‐depleted fetal bovine serum (Exo‐FBS, System Biosciences, California, USA), 100 U mL^−1^ penicillin, and 100 µg mL^−1^ streptomycin (Thermo Fisher Scientific, Massachusetts, USA). The collected supernatants were centrifugated at 2000 x g for 10 min;10 000 x g for 60 min, and filtrated through a 0.22 µm pore‐size filter (MF‐Millipore) to sequentially remove large vesicles, smaller apoptotic bodies, and microvesicles. The filtrate was then ultracentrifuged at 100 000 x g for 90 min twice at 4 °C to pellet the exosomes and gently resuspended in 1xPBS. The final exosome was quantified using the BCA Protein Assay Kit (Vazyme Biotech, Nanjing, China). Exosomes were visualized by an electron microscopy (TEM, Hitachi HT7800, Tokyo, Japan) and the size contribution was determined by a Dynamic Light Scattering (Malvern Instruments, Malvern, UK). The expression of exosomal markers Alix, CD63, and negative marker GAPDH (Proteintech, Chicago, USA) were tested by western blotting analysis.

### Exosomes Tracking and Distribution

For tracking exosomes throughout the entire eyeball after subconjunctival injection (SCI), exosomes were labeled with Dio (Green fluorescent, Sangon Biotech, Shanghai, China). Exosomes initially were diluted in 1 mL PBS and stained with 5 µL Dio (2×10^6^ m) for 20 min at room temperature, Unincorporated Dio was removed by ultracentrifuge at 100 000xg for 60 min. The Dio‐labeled exosomes were injected subconjunctivally to trace the distribution in graft‐bearing ocular tissues. On day 6 post‐transplantation, the entire eyeball with conjunctiva was excised and OCT‐embedded cryosections (10 µm) were obtained, mounted with Fluoroshield with DAPI histology mounting medium (Sigma–Aldrich, Saint Louis, USA).

### Murine and Corneal Transplantation

All mice used in this study were housed in a specific pathogen‐free facility. Male BALB/C and C57BL/6 mice, aged six to 8 weeks, were purchased from Hunan Slake Jingda Experimental Animals Co. Ltd in China. For allogenic and isogenic transplantation procedures, BALB/C mice were used as the recipients, while C57BL/6 and BALB/C both served as the donors respectively. The corneal transplantation was performed in adherence to previously published methods. After donor mice were euthanized via cervical dislocation, a full‐thickness cornea section (2.25 mm in diameter) was carefully excised. Before accepting transplantation, recipient mice were anesthetized through an intraperitoneal injection of 16.5 mg mL^−1^ pentobarbital sodium. The corneal graft cut out from the donor, was then sutured onto the 2 mm diameter graft bed of the recipient using 11‐0 nylon sutures. Additionally, tarsorrhaphy was performed immediately using 8‐0 nylon sutures with needles. Postoperative care involved the removal of sutures from the tarsal area and cornea on the 3rd and 7th day post‐transplantation, respectively. All graft conditions were monitored and assessed under a slit‐lamp biomicroscopy system (Leicca, Weztlar, Germany) every other day from day 7 to day 42. The observer was blinded to the experiment design. One graft was considered rejected when it presented a stromal opacity (i.e., invisible iris texture and pupillary margin).^[^ ^17^ ^]^ For JAK2 inhibitor treatment, mice underwent subconjunctival injections of 2 µg of B16‐Exo or JB6‐Exo in 50 µL of PBS weekly until the end of the observation period. AG490 (Selleck, California, USA), a JAK2 inhibitor, was dissolved in dimethyl sulfoxide as per manufacturer's instructions and administered intraperitoneally at a dose of 5 mg kg^−1^ daily for 25 days. All animal procedures were performed according to the animal ethical guidelines and approved by the Experimental Animal Center of Central South University of China (File No. 20231195).

### Quantitative Analysis of Corneal Neovascularization

The quantification of neovascularization within the corneal grafts was conducted using immunofluorescence staining to evaluate the emergence of new blood vessels. On day 18 post‐transplant, the corneas from eye ball‐bearing grafts were excised and fixed in acetone for 20 min. To minimize non‐specific binding, the fixed corneas were blocked by PBS containing 10% donkey serum (Absin Bioscience, Shanghai, China) for 1 h. For the identification of corneal blood vessels, the tissues were incubated overnight at 4 °C with a primary antibody specifically targeting CD31 (R&D Systems, Minnesota, USA) and then incubated with a specific secondary antibody Alexa‐Flour 594 (Jackson, Pennsylvania, USA) for 2 h at room temperature. Post‐staining, the corneas were mounted using Fluoroshield with DAPI histology mounting medium (Sigma–Aldrich, Saint Louis, USA), preserving fluorescence and nuclear counterstaining. The mounted samples were protected from light at 4 °C till imaging. The percentage of neovessels perfusion area to the entire cornea was quantified with Image J (NIH Image J system, 1.8.0, Bethesda, USA).

### In Vitro Angiogenesis Assay

Initially, Matrigel was thawed overnight at 4 °C, while 24‐well plates, 200 µL pipette tips, and 1 mL Eppendorf tubes were pre‐chilled to −20 °C to maintain the integrity of the matrix. The following day, the Matrigel was promptly transferred from the refrigerator to pre‐chilled Eppendorf tubes, using pre‐chilled pipette tips, 200 µL of Matrigel was uniformly dispensed into each well of the 24‐well plate. After application, the plate was incubated at 37 °C for 30 min to allow complete gelation of the Matrigel. Endothelial cells were prepared as a single‐cell suspension and counted. Once the Matrigel had solidified, the cell suspension was adjusted to a density of 1 × 10^5^ cells mL^−1^ and gently added to each well. After seeding, Vascular endothelial growth factor (VEGF) was added at a concentration of 30 ng mL^−1^ to stimulate angiogenesis. Additionally, B16‐Exo and JB6‐Exo were supplemented at 60 µg mL^−1^ to evaluate their inbitition effects on vessel formation. The cells were cultured at 37 °C with 5% CO^2^ for 8 h. Following the incubation, angiogenesis was assessed under an inverted microscope. New vessel formation was quantified by randomly selecting five fields per well. The lengths of the newly formed vessels were measured, and quantified using ImageJ software.

### Histology and Immunohistochemistry

Hematoxylin and eosin (H&E) staining was performed to determine the degree of corneal irregularity, corneal edema, and inflammatory cell infiltration. CD45^+^ and CD3^+^ cell infiltration staining, indicative of immune cell and T cell presence respectively, was conducted. On day 18 post‐transplant, the entire eyeball bearing the graft was fixed in 4% formaldehyde, and then embedded in paraffin prepared for both H&E and immunohistochemical staining.

Paraffin‐embedded sections were dewaxed and dehydrated first. Then the sections were placed in a repair cassette filled with EDTA antigen repair buffer (Servicebio, Wuhan, China) to repair the antigen in a microwave oven. Endogenous peroxidase was blocked with 3% hydrogen peroxide for 15 min and proteins were blocked in serum‐blocking buffer (10% goat serum, 1% BSA, 22 mg mL^−1^ glycine, 0.1% Tween‐20) for 1 h. After incubated in the primary antibody (Abcam Bioscience, Cambridge, US) at 4 °C overnight, the slides were incubated with HRP secondary antibody (AiFang biological, Hunan, China) at room temperature for 40 min protected from light. The fluorescent dyes TYR‐570 and TYR‐520 were utilized for the visualization of CD45^+^ and CD3^+^ cells respectively. The slides were sealed with Fluoroshield with DAPI histology mounting medium (Sigma–Aldrich, Saint Louis, USA) and photographed under the microscope (Carl Zeiss, Axio Imager M2+Apotome 3, Oberkochen, Germany).

### Bone Marrow Cells and MDSC Isolation

Bone marrow cells were isolated from the marrow of Balb/c mice and cultured in RPMI 1640 medium with 10% fetal bovine serum (FBS; Gibco, Australia), 100U mL^−1^ of penicillin, and 100 µg mL^−1^ of streptomycin. To specifically stimulate the population of myeloid‐derived suppressor cells (MDSC) within the marrow cell cultures, the 10 ng mL^−1^ of granulocyte‐macrophage colony‐stimulating factor (GM‐CSF) and 10 ng mL^−1^ of interleukin 6 (IL‐6) (Perprotech, Rocky Hill, USA) were added in culture medium.

### Flow Cytometric Analysis of MDSC Subpopulations

Flow cytometry was used to assess MDSC levels in mice tissues post corneal transplantation or in vitro MDSC culture. Single‐cell suspensions were obtained from bone marrow, blood, and spleen on day 18 post corneal transplantation. After lysis of red blood cells (BD, New Jersey, USA), isolated cells were stained with Zombie Aqua fixable viability dye (BioLegend, California, USA) to stain dead cells. All antibodies were purchased from BD Biosciences (BD, New Jersey, USA). Anti‐CD16/32 antibody was used to block the Fc receptor before all surface staining. Then MDSC were recognized by APC‐Cy7 CD45 antibody, APC CD11b antibody, and PE granulocyte‐differentiation antigen 1 (Gr‐1) antibody. FITC lymphocyte antigen 6 complex, locus C (Ly6C) antibody, and PerCP‐Cy5.5 lymphocyte antigen 6 complex, locus G (Ly6G) antibody were used to identify MDSC subsets, i.e. monocytic MDSC (M‐MDSC) and polymorphonuclear MDSC (PMN‐MDSC). Total MDSC, M‐MDSC, and PMN‐MDSC were recognized by CD45^+^CD11b^+^Gr‐1^+^, CD45^+^CD11b^+^Gr‐1^+^Ly6G^−^Ly6C^hi^, and CD45^+^CD11b^+^ Gr‐1^+^Ly6G^+^Ly6C^lo^ cell populations respectively.

### Cytokines Quantification

The cytokines (IL‐6, IL‐2, IL‐4, IFN‐γ, TNF, IL‐17A, and IL‐10) level in mice blood serum was detected using BD Cytometric Bead Array Kit (BD, New Jersey, USA). The procedure was according to the manufacturer's instructions. After beads coated with specific antibodies captured the cytokine molecules present in the serum, the bound beads were then detected and quantified using the Northern Lights Flow Cytometry Instrument (CYTEK NL‐3000, USA).

### Proteomic Analysis of Exosomes

Exosome samples were mixed with an appropriate volume of lysis buffer and heated in a boiling water bath for 15 min and then centrifuged at 14 000 g for 15 min. The supernatant was collected. Protein concentration was quantified using the BCA method and stored at −80 °C. For each sample, 20 µg of protein was mixed with 6X loading buffer and boiled for 5 min, followed by 12% SDS‐PAGE electrophoresis at a constant voltage of 250 V for 40 min, and the gels were stained with Coomassie Brilliant Blue. For mass spectrometry preparation, 100 µg of protein solution from each sample was treated with TCEP to a final concentration of 10 mm and heated again for 10 min, then cooled to room temperature. CAA was added to a final concentration of 40 mm, and the mixture was incubated in the dark at room temperature for 30 min. Then, 100 µL of 50 mm NH_4_HCO_3_ solution and 40 µL of trypsin buffer (4 µg trypsin in 40 µL 50 mm NH_4_HCO_3_ solution) were added. The samples were agitated at 600 rpm for 1 min and incubated at 37 °C for 16–18 h. Subsequently, an appropriate amount of 20% TFA was added to reach a final concentration of 0.5%, followed by centrifugation at 14 000 g for 15 min. The supernatant was desalted using C18 Cartridges. The desalted peptides were lyophilized and reconstituted in 40 µL of 0.1% formic acid for peptide quantification (OD280 measurement). For mass spectrometry analysis, samples were separated using a nano‐flow rate on a NanoElute system, connected to a timsTOF Pro mass spectrometer equipped with a CaptiveSpray ion source (Bruker, Bremen, Germany). Buffer A was 0.1% formic acid in water, and buffer B was 0.1% formic acid in acetonitrile (100% acetonitrile). The chromatographic column was equilibrated with 100% buffer A, and samples were loaded onto the analytical column (IonOpticks, Australia, 25 cm x 75 µm, C18 packing 1.6 µm) by an autosampler at a flow rate of 300 nL min^−1^. Post‐separation, the samples were analyzed by the PASEF mode of the timsTOF Pro mass spectrometer. The ionization mode was set to positive, with a parent ion scan range of 100–1700 m/z and ion mobility range of 0.75–1.4 V⋅s cm^−2^. Ion accumulation or release time was 100 ms, ion utilization rate was 100%, capillary voltage was set at 1500 V, dry gas flow rate at 3 L min^−1^, and drying temperature at 180 °C. The PASEF settings were as follows: ten MS/MS scans per cycle (total cycle time of 1.16 s), charge range from 0 to 5, dynamic exclusion duration of 0.5 min, ion target intensity of 10 000, ion intensity threshold of 2500, and CID fragmentation energy range.

### CD4^+^ T Cells Proliferation Assay

The immunosuppressive capacity of MDSC was evaluated based on their ability to inhibit the proliferation of CD4^+^ T cells. CD4^+^ T cells were isolated from the spleen of Balb/c with CD4 (L3T4) microbeads (Miltenyi Biotec, Nordrhein‐Westfalen, Germany). CD4^+^ T cells were then pre‐stained by carboxyfluorescein succinimidyl amino ester (2.5 µm, Invitrogen, California, USA). Cells were cultured in RPMI‐1640 (Gibco, Shanghai, China) supplemented with 10% fetal bovine serum, 1% HEPES buffer (LIFE technologist, New Mexico, USA), 1% sodium pyruvate (LIFE technologist, New Mexico, USA), and 1% nonessential amino acids (LIFE technologist, New Mexico, USA). Stimulation of the CD4^+^ T cells was achieved using precoated plates with 5 µg mL^−1^ of anti‐CD3 and 2 µg mL^−1^ of anti‐CD28 antibodies. Equal MDSC were cocultured with CD4^+^ T cells (1 × 10^5^ well^−1^) in a 96‐well round‐button plate (Corning, New York, USA). After 5 days, CD4^+^ T cells were further stained with PE‐conjugated CD4 antibody. The proliferation rate was detected using the Northern Lights Flow Cytometry Instrument.

### Cell Viability Assay

MDSC viability was assessed using the CCK‐8 assay (Biosharp, Anhui, China). MDSCs treated with B16‐Exo (60 µg mL^−1^) were incubated in 96‐well plates for 24, 48, and 72 h. CCK8 solution (10% v/v in culture medium) was added, and the cells were incubated for 3 h at 37 °C. Absorbance at 450 nm was measured to determine cell proliferation.

### Flow Cytometric Analysis of MDSC Subpopulations Post‐Corneal Transplantation

Flow cytometry was employed to quantify MDSC and Treg populations in murine tissues following corneal transplantation and in vitro cultures. Single‐cell suspensions were prepared from bone marrow, blood, cervical lymph nodes, and spleens at day 18 post‐transplantation. Red blood cells were lysed using a commercial lysis buffer (BD Biosciences, New Jersey, USA). Cell viability was assessed using Zombie Aqua fixable viability dye (BioLegend, California, USA). Non‐specific Fc receptor binding was blocked using anti‐CD16/32 antibody (BD Biosciences, New Jersey, USA) prior to staining. MDSCs were identified with APC‐Cy7‐labeled CD45, APC‐labeled CD11b, and PE‐labeled Gr‐1 antibodies. Monocytic MDSCs (M‐MDSC) and polymorphonuclear MDSCs (PMN‐MDSC) were further differentiated using FITC‐labeled Ly6C and PerCP‐Cy5.5‐labeled Ly6G antibodies, respectively. Tregs were detected using PE‐CF594‐labeled CD4, BB515‐labeled CD25, and AF647‐labeled Foxp3 antibodies, identifying cells as CD4^+^CD25^+^Foxp3^+^.

### Statistical Analysis

The statistical analysis of the data collected in this study was conducted using SPSS software, version 20 (SPSS Inc., Chicago, USA). Corneal graft survival was plotted according to the Kaplan–Meier method and compared by log‐rank test. A one‐way analysis of variance (ANOVA) with Least Significance Difference (LSD) post‐test was used for multiple group comparisons. All data were visualized with GraphPad Prism 8.4.0 (San Diego, CA, USA.).

## Conflict of Interest

The authors declare no conflict of interest.

## Supporting information



Supporting Information

## Data Availability

The data that support the findings of this study are available from the corresponding author upon reasonable request.
