# Supplementary material for: Harnessing Tumor Cell‐Derived Exosomes for Immune Rejection Management in Corneal Transplantation
Source: Adv Sci (Weinh). 2024 Nov 14;12(2):2409207. doi: 10.1002/advs.202409207 (PMC11727379; doi:10.1002/advs.202409207)
Supplement: Supplementary file 1 — Supporting Information [file ADVS-12-2409207-s001.docx]

Supporting Information

Harnessing Tumor Cell-Derived Exosomes for Immune Rejection Management in Organ Transplantation

Jieru Yang^1#^, Huanmin Kang^2#^, Yingyi Liu^3^, Shan Lu^1^ Huihui Wu^4^, Bikui Zhang^4^, Yan He^3*^, Wenhu Zhou^1,5*^

^1^Xiangya School of Pharmaceutical Sciences, Central South University, Changsha, Hunan 410013, China.

^2^Department of Ophthalmology, West China Hospital, Sichuan University, Chengdu, Sichuan 610041, China.

^3^Beijing Tongren Eye Center, Beijing Tongren Hospital, Capital Medical University; Beijing Key Laboratory of Ophthalmology & Visual Sciences, Beijing 100730, China

^4^ Department of Pharmacy, The Second Xiangya Hospital, Central South University, Changsha, China.

^5^Key Laboratory of Biological Nanotechnology of National Health Commission, Changsha City, 410008, Hunan Province, P.R. China.

Email：[zhouwenhuyaoji@163.com](mailto:zhouwenhuyaoji@163.com); [dryanhe@163.com](mailto:dryanhe@163.com)

^#^Jieru Yang and Huanmin Kang contributed equally to this work

Table S1.

| Antibody and cell culture medium | Vendor | Catalog No. | Dilution or concentration |
| --- | --- | --- | --- |
| Flow Cytometry |  |  |  |
| anti-mouse CD16/32 | Biolegend, San Diego, CA, USA | 156604 | 1:200 |
| PE anti-mouse CD4 | Biolegend, San Diego, CA, USA | 100408 | 1:100 |
| CellTrace CFSE Cell Proliferation Kit | Invitrogen, Carlsbad, California, USA | C34554 | 2.5μM |
| APC-Cy7 rat anti-mouse CD45 antibody | BD Biosciences, San Diego, CA, USA | 103116 | 1:100 |
| APC rat anti-mouse CD11b antibody | BD Biosciences, San Diego, CA, USA | 553312 | 1:100 |
| PE rat anti-mouse Gr-1 antibody | BD Biosciences, San Diego, CA, USA | 553128 | 1:100 |
| FITC rat anti-mouse Ly6C antibody | BD Biosciences, San Diego, CA, USA | 553104 | 1:100 |
| PerCP-Cy5.5 rat anti-mouse Ly6G antibody | BD Biosciences, San Diego, CA, USA | 560602 | 1:100 |
| BD Cytometric Bead Array Kit | BD Biosciences, San Diego, CA, USA | 560485 | According manufacture's insturctions |
| Immunofluorescent staining |  |  |  |
| Goat anti-mouse CD31 antibody | R&D Systems, Minneapolis, MN, USA | AF3628 | 1:100 |
| Rabbit anti-mouse LYVE-1 antibody | Abcam Bioscience, Cambridge, MA, USA | Ab14917 | 1:100 |
| Alexa Fluor 488-conjugated AffiniPure donkey anti-rabbit IgG (H+L) secondary antibody | Jackson ImmunoResearch Laboratories, West Grove, PA, USA | 711-545-152 | 1:100 |
| Alexa Fluor 594-conjugated donkey anti-goat IgG (H+L) secondary antibody | Jackson ImmunoResearch Laboratories, West Grove, PA, USA | 705-585-003 | 1:100 |
| Immunohistochemistry staining |  |  |  |
| EDTA antigen repair buffer | Servicebio, Wuhan, China | G1203 | According manufacture's insturctions |
| Anti-CD45 antibody | Abcam Bioscience, Cambridge, MA, USA | ab208022 | 1:3000 |
| Anti- CD3 antibody | Abcam Bioscience, Cambridge, MA, USA | ab135372 | 1:3000 |
| HRP secondary antibody | AiFang biological, Hunan, China | AFIHC003 | According manufacture's insturctions |
| Mutiplex immunofluorescence staining kit | AiFang biological, Hunan, China | AFIHC024 | According manufacture's insturctions |
| MDSC Cells culture medium |  |  |  |
| Basic DMEM/F-12 Medium | Gibco, Shanghai, China | C11330 |  |
| Fetal bovine serum | Gibco, Australia | 10099141C | 10% |
| Recombinant Murine GM-CSF | PreproTech, Rocky Hill, USA | 315-03 | 10ng/ml |
| Recombinant Murine IL-6 | PreproTech, Rocky Hill, USA | 216-16 | 10ng/ml |
| CD4+ T cell proliferation culture medium |  |  |  |
| Basic RPMI 1640 Medium | Gibco, Shanghai, China | C11875 |  |
| Fetal bovine serum | Gibco, Australia | 10099141C | 10% |
| Sodium pyruvate | LIFE technologist, Karlsbad, NM, USA | 11360070 | 1% |
| HEPES buffer | LIFE technologist, Karlsbad, NM, USA | 15630080 | 1% |
| Nonessential amino acids | LIFE technologist, Karlsbad, NM, USA | 11140050 | 1% |
| CD3e Monoclonal Antibody | eBioscience, San Diego, CA, USA | 16-0031-81 | 10 mg/mL |
| CD28 Monoclonal Antibody | eBioscience, San Diego, CA, USA | 16-0281-81 | 5 mg/mL |
| Western Blotting |  |  |  |
| Alix antibody | Proteintech, Chicago, USA | 12422-1-AP | 1:10000 |
| CD63 antibody | Proteintech, Chicago, USA | 25682-1-AP | 1:1000 |
| GAPDH antibody | Proteintech, Chicago, USA | 10494-1-AP | 1:1000 |
| HRP-conjugated Goat Anti-Rabbit IgG | Proteintech, Chicago, USA | SA00001-2 | 1:10000 |
| Others |  |  |  |
| 3,3'-Dioctadecyloxacarbocyanine perchlorate | MedChemExpress, New Jersey, USA | 34215-57-1 | According manufacture's insturctions |
| AG-490 (Tyrphostin B42) | Selleck Chemicals, Houston, USA | S1143 | 50 µM |
| Fluoroshield with DAPI-histology mounting medium | Sigma-Aldrich, St. Louis, USA | F6057 | According manufacture's insturctions |


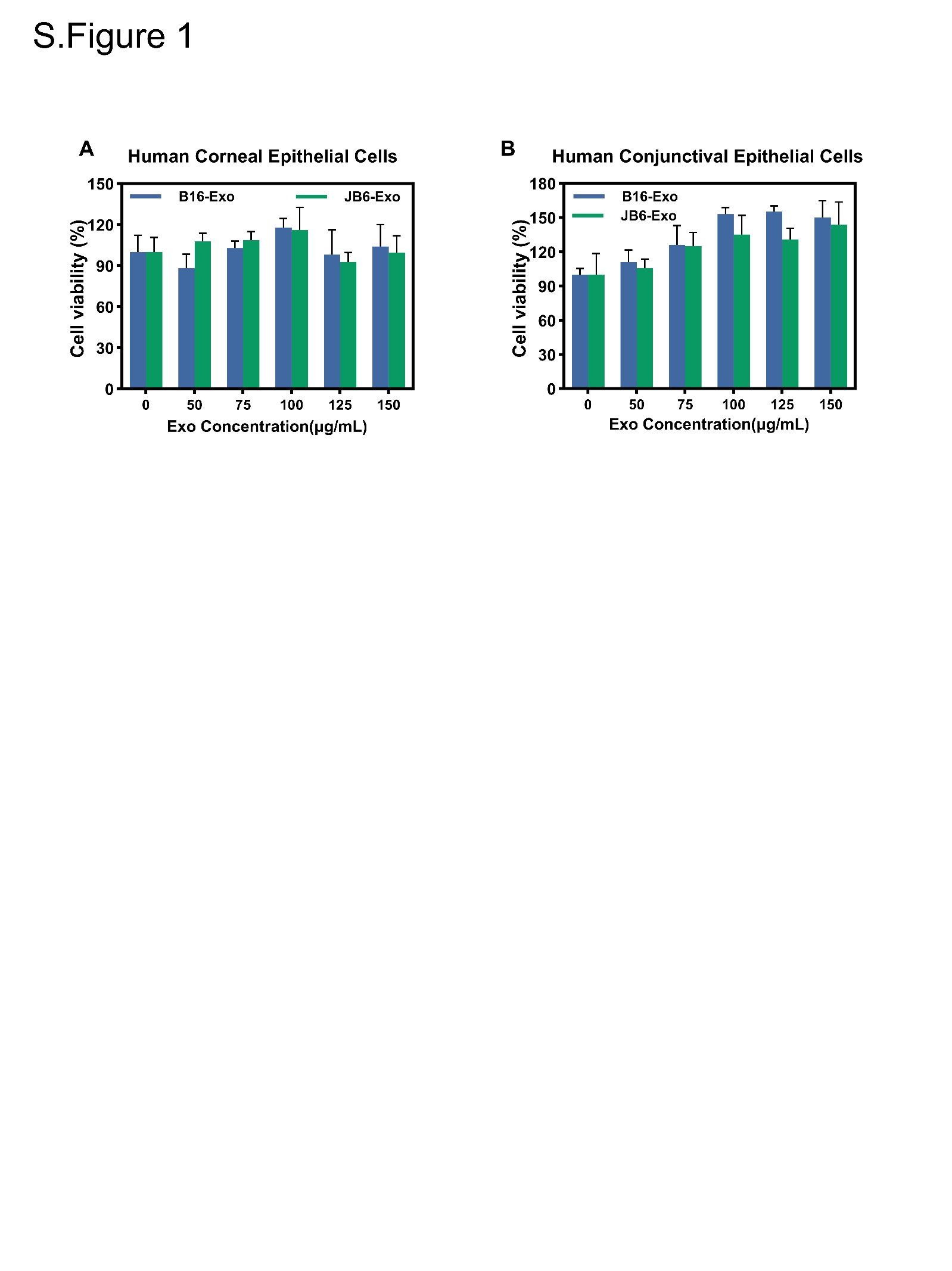


**Figure S1.** Cytotoxicity assay of B16-Exo on corneal epithelial and conjunctival epithelial cells. data represent mean ± SD of three independent experiments.


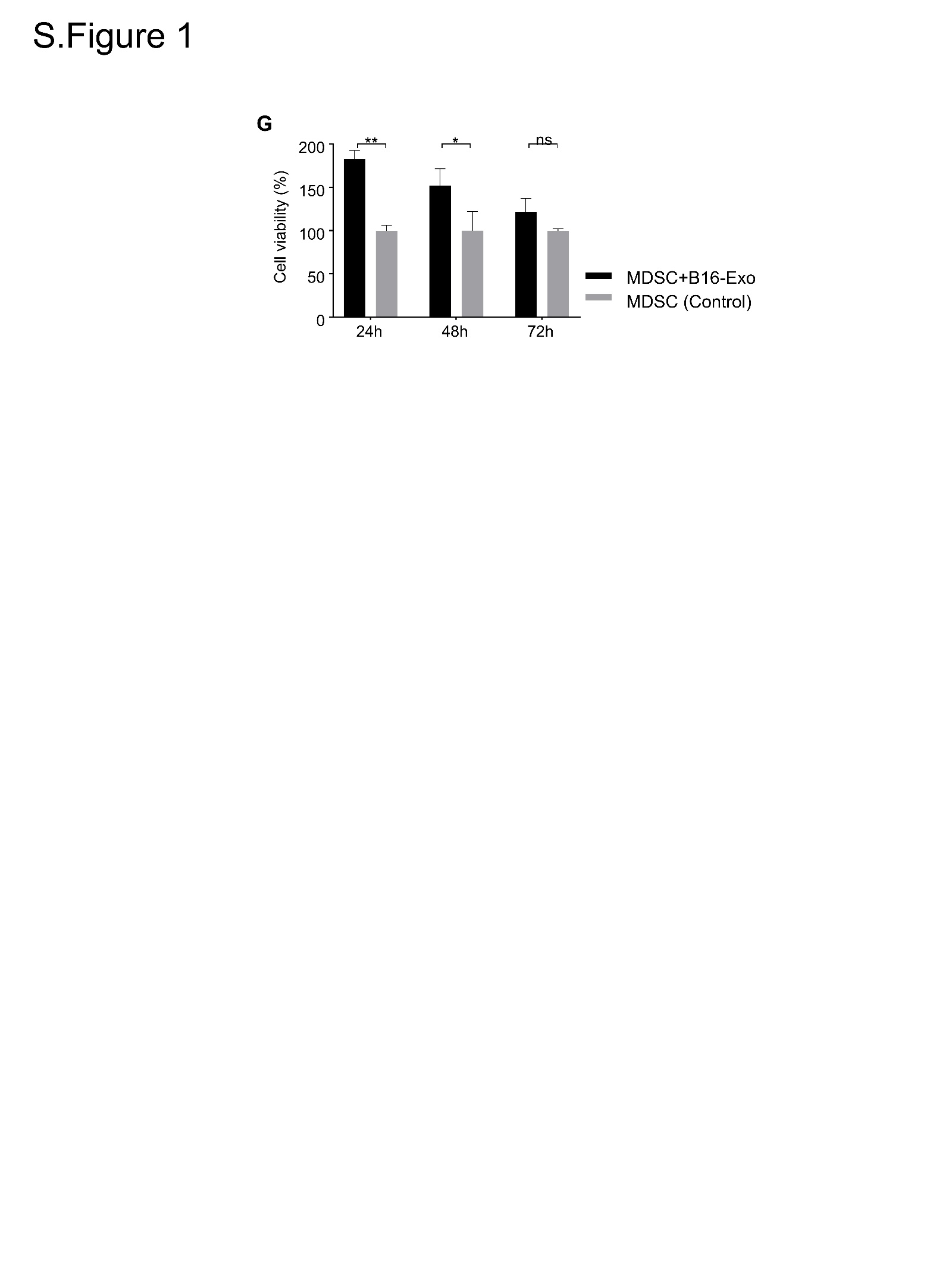


**Figure S2.** Effects of B16-Exo on MDSC Viability Assessed by CCK-8 Assay, data represent mean ± SD of three independent experiments.


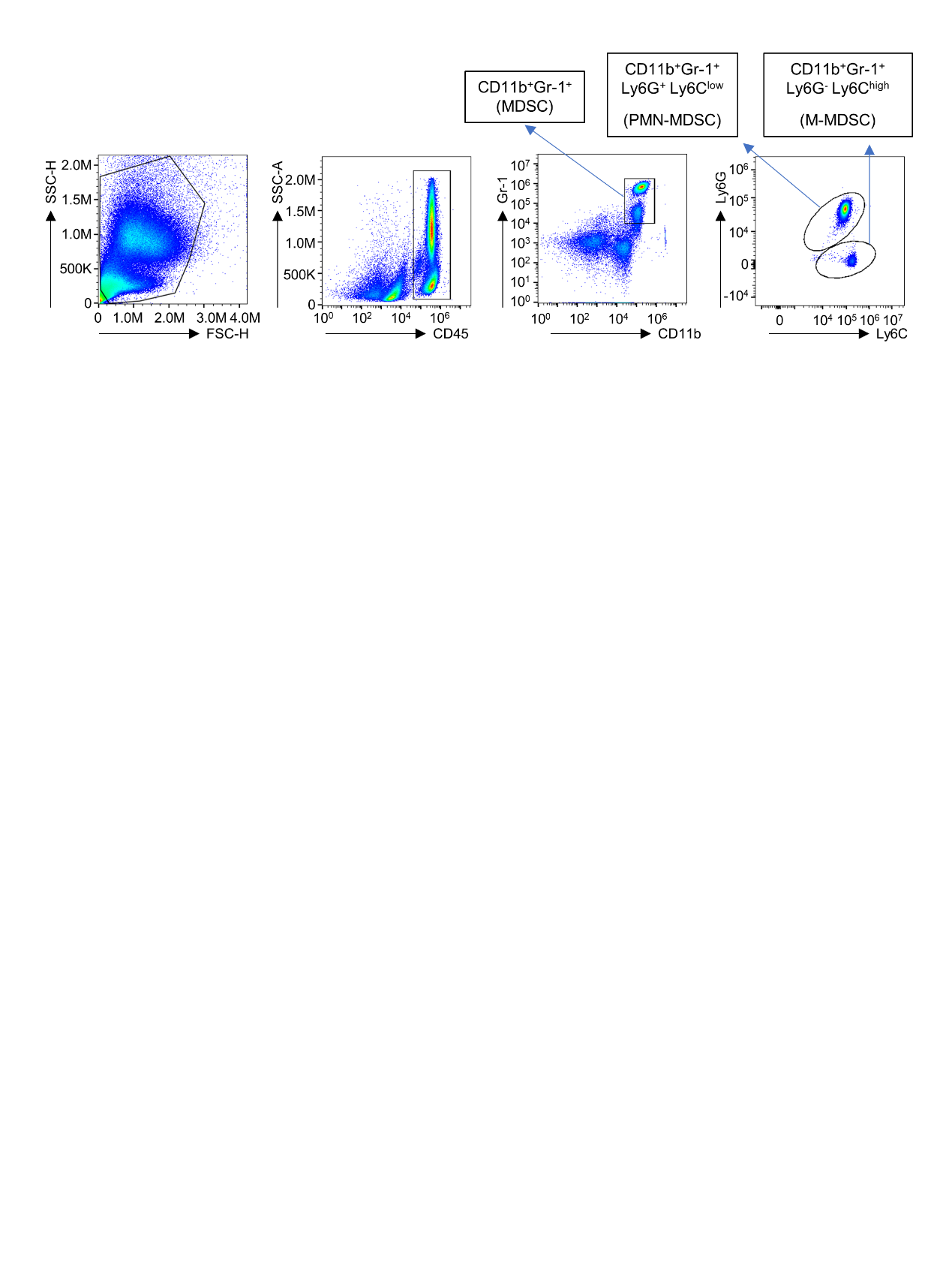


Figure S3. Representative flow cytometry cytograms of MDSC (CD11b+Gr-1+), PMN-MDSC (CD11b+Gr-1+Ly6G+Ly6Clo) and M-MDSC(CD11b+Gr-1+Ly6G-Ly6Chi) on day 18 posttransplant.


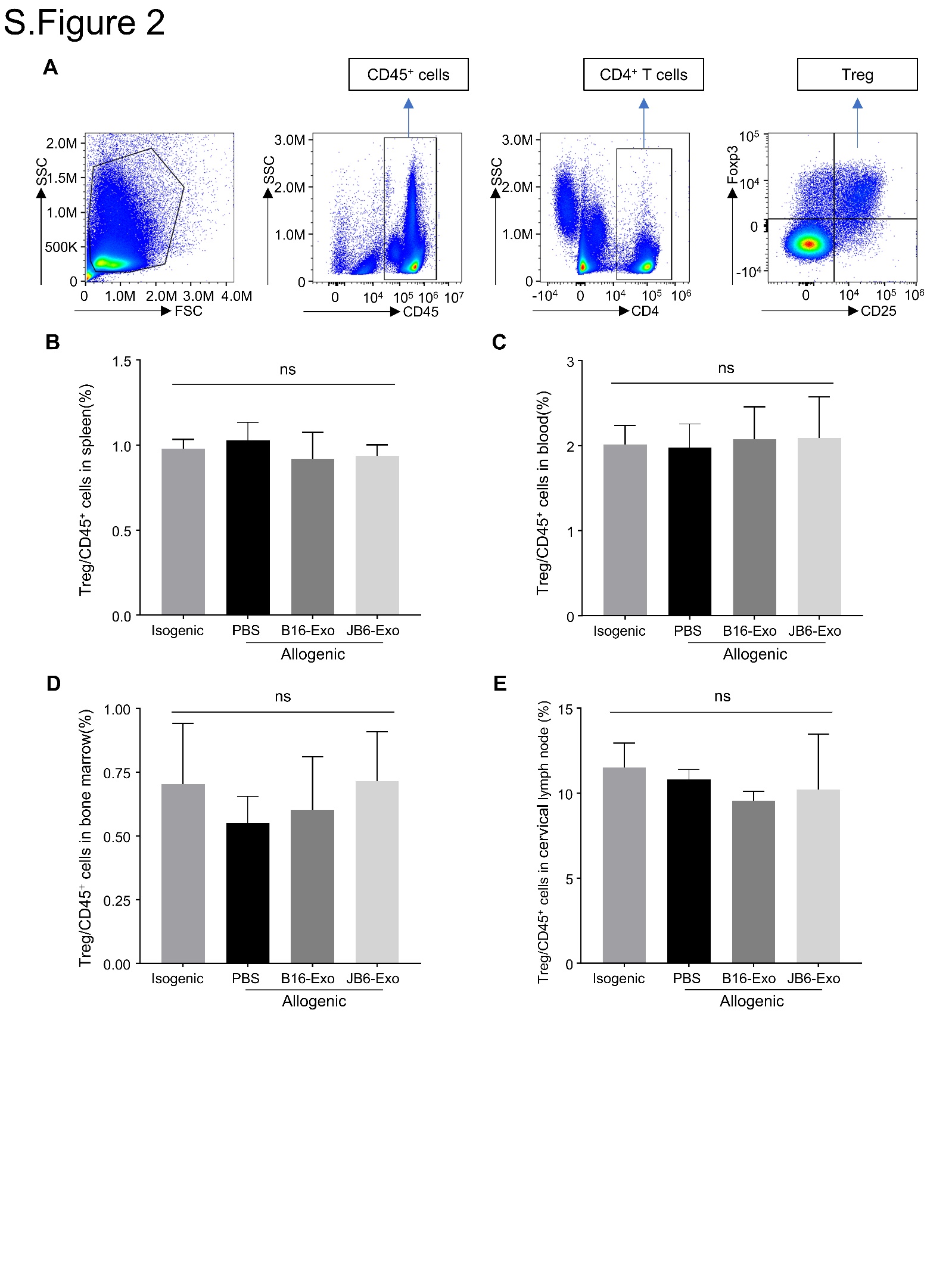


Figure S4. (A) Flow cytometry gating strategy used to identify CD45^+^ cells, CD4^+^ T cells, and Tregs (CD4^+^CD25^+^FoxP3^+^) from mouse tissues on day 18 posttransplant. (B-E) Quantitative analysis of Treg populations in the spleen (B), blood (C), bone marrow (D), and cervical lymph nodes (E).


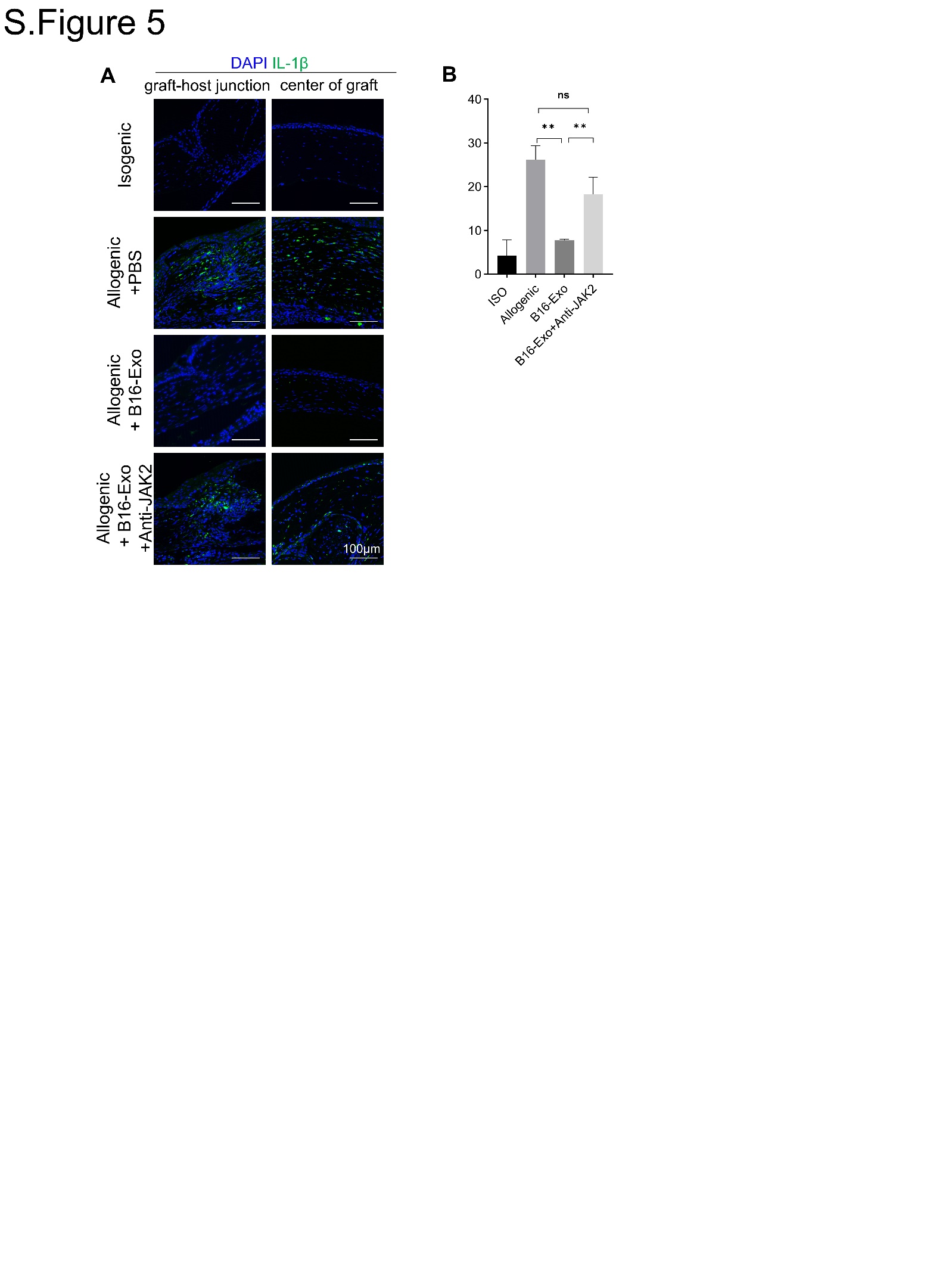


**Figure S5**. (A) The Immunofluorescence of IL-1β in corneal transplants, IL-1β staining (green) at the graft-host junction and the center of the graft; (B) Quantitative analysis of IL-1β expression levels. Data are shown as mean ± SEM.
